# The mitochondrial pyruvate carrier as a mediator of the Warburg Effect and its impact on cancer growth and metabolism

**DOI:** 10.1186/2049-3002-2-S1-O14

**Published:** 2014-05-28

**Authors:** John Schell, Kristofor Olson, Jared Rutter

**Affiliations:** 1 Department of Biochemistry, University of Utah School of Medicine, Salt Lake City, Utah, USA

## Background

The step between pyruvate production by glycolysis in the cytosol and movement into the mitochondrial matrix has largely been unstudied due to the lack of identity of the genes that mediate this process. The discovery of the Mitochondrial Pyruvate Carrier (MPC) opens this overlooked aspect of metabolism to interrogation. The dispensation of pyruvate represents an important and highly regulated decision within the cell. This is especially true in rapidly proliferating cells including those found in tumors. Given the reliance of cancer cells on glycolysis and the concomitant suppression of mitochondrial metabolism we hypothesize that modulation of the MPC may serve to promote and reinforce the Warburg Effect.

## Materials and methods

Publicly available datasets were queried for differential expression of MPC1 and MPC2 (TGCA and GEO). An array of cell lines representing various cancers were stably infected with MPC1 and MPC2 or empty vector and assayed for growth differences in 2D as well as 3D (soft agar and spheroid formation).

## Results

MPC1 and MPC2 transcript levels are significantly reduced in multiple cancers compared to the adjacent normal tissue including forms of gastric, colon, breast, ovarian, and renal cancers. These expression changes have been confirmed using qPCR and immunoblotting. In addition to altered expression, low MPC correlates with decreased survival across multiple cancer types. We reasoned that cancer cells would reduce MPC expression to promote the Warburg Effect to fuel rapid growth and relied on *in vitro* cell culture to manipulate MPC expression. The re-expression of MPC1 and MPC2 results in no change in cell viability or apoptosis but causes reduced attachment in culture plates as well as reduced growth in soft agar and spheroids. In addition to the *in vitro* studies, xenograft models have recapitulated these growth differences. We have confirmed MPC expression produces alterations in metabolism including increased oxygen consumption with pyruvate as a substrate, a reduction in lactate production, and an increase in mitochondrial membrane potential.

## Conclusions

MPC expression is reduced in many types of cancer and its re-expression results in changes in metabolism and growth as measured by multiple assays. As expected, MPC expression facilitates pyruvate movement into the mitochondria and reduces the Warburg Effect. We are now investigating the effect of MPC expression on different cell subpopulations as well as the root cause of the expression changes observed in cancer.

